# sparse-growth-curve: a Computational Pipeline for Parsing Cellular Growth Curves with Low Temporal Resolution

**DOI:** 10.1128/MRA.00296-21

**Published:** 2021-05-13

**Authors:** Chuankai Cheng, J. Cameron Thrash

**Affiliations:** a Department of Biological Sciences, University of Southern California, Los Angeles, California, USA; Indiana University, Bloomington

## Abstract

Here, we introduce a Python-based repository, sparse-growth-curve, a software package designed for parsing cellular growth curves with low temporal resolution. The repository uses cell density and time data as the input, automatically separates different growth phases, calculates the exponential growth rates, and produces multiple graphs to aid in interpretation.

## Abstract (precis)

Here, we introduce a Python-based repository sparse-growth-curve, a software package designed for parsing cellular growth curves with low temporal resolution. The repository uses cell density and time data as the input, automatically separates different growth phases, calculates the exponential growth rates, and produces multiple graphs to aid in interpretation.

## ANNOUNCEMENT

The cellular growth curve arising from cell density variation over time is an important phenotype for cell physiology and systems biology studies (1). Improving the throughput of growth rate calculations from growth curves benefits from automated curve-fitting algorithms, but most existing software has been developed for data with high temporal resolution (2–8). Such data are usually generated by automated optical density measurements (5), which apply only to a limited set of organisms that can achieve high cell densities. Absolute cell count quantification (cells per milliliter) can be labor-intensive for high-temporal-resolution growth curves when methods like flow cytometry or microscopy are used. Therefore, we have developed a growth-curve-fitting pipeline specifically for characterizing sparse data.

**FIG 1:**
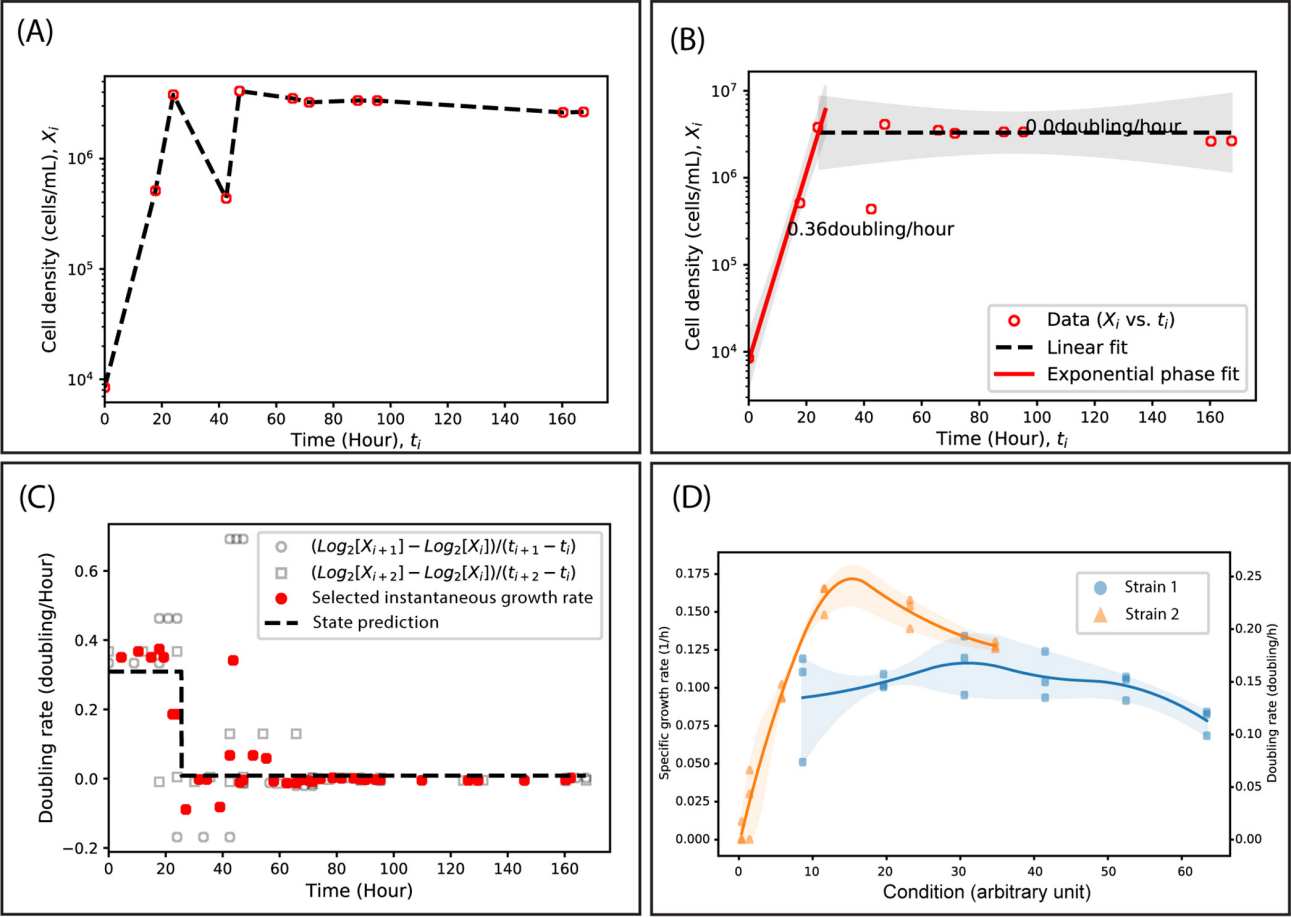
Overview of the process within sparse-growth-curve. (A) The input for the method (red open circles connected by dashed lines) is cell density *X_i_* versus time *t_i_*. Here, we use an imperfect and very sparse curve to illustrate the functionality of the software. (B) The output of the method is piecewise linear regression for each phase. The exponential phase is highlighted in red. The doubling rates are labeled. (C) The subdomains of the piecewise fit in plot B are determined through decision tree regression. Here, gray open circles and squares represent the instantaneous doubling rates calculated from the input data. Red solid squares represent the noise-removed rates versus time for decision tree regression. The state prediction (dashed line) ends up as a step function, and each step corresponds to a growth phase. (D) An extended feature of the sparse-growth-curve package is that the package imports experimental growth curves of multiple strains under multiple conditions. Exponential growth rates for all of the growth curves are calculated. The conversion of doubling rate γ to specific growth rate λ is λ = ln(2)γ. The solid lines are the interpolated connection mean growth rates, and the shaded areas indicate the range between the maximum and minimum growth rates.

The sparse-growth-curve method takes an array of cell density *X_i_* and the corresponding acquisition time *t_i_* (see the template Excel file at GitHub, https://github.com/thrash-lab/sparse-growth-curve) as the input (i.e., typical growth data) (Fig. 1A). The method differentiates growth phases and returns a piecewise linear fit on a logarithmic scale (Fig. 1B). The growth phases are determined through decision tree regression (Fig. 1C), based on defining each growth phase as a period with similar instantaneous growth rates (see https://github.com/thrash-lab/sparse-growth-curve/blob/main/detailed_method.md for detailed methods). The result is automated detection of exponential phase in sparse data and calculation of growth rates according to linear regression.

The software package can analyze multiple growth curves from different replicates, strains, or conditions (e.g., salinity, temperature, or substrate). The output includes multiple products, including (i) plots of the raw growth data on a logarithmic scale, including the best-fit linear regressions for exponential phase; (ii) box plots of growth rates separated by treatment and colored by replicates; (iii) growth rate calculations in both doubling and specific growth rates, with output in an Excel file; and (iv) multistrain/replicate plots of growth rates for experimental designs with continuous variables (e.g., temperature) (Fig. 1D).

### Data availability.

sparse-growth-curve is available on GitHub (https://github.com/thrash-lab/sparse-growth-curve) under MIT license. The code can be run directly in the browser through Google Colaboratory (https://colab.research.google.com/notebooks/intro.ipynb) without any configuration required on a local machine. The code in iPython Notebook v5.5.0 format illustrates the detailed steps of the methods. There are three separate notebooks, i.e., (i) a notebook showing how a single growth curve (*X_i_* versus *t_i_*) (Fig. 1A) is fitted (Fig. 1B); (ii) a notebook showing how a single Excel file with multiple growth curves is parsed and characterized; and (iii) a notebook that can handle multiple Excel files (people may want to put growth curves collected from different experimental runs in separate files) and analyze them all in a batch. The Python v3.7.10 (9) code relies on multiple packages (which are automatically installed by running the script in the Google Colab notebooks), including NumPy v1.19.5 (10) for numerical analysis, SciPy v1.4.1 (11) and Scikit-learn v0.22.2 (12) for statistical analysis, Pandas v1.1.5 (13) for handling table format data, Matplotlib v3.2.2 (14) for data visualization, and XlsxWriter v1.3.9 (https://github.com/jmcnamara/XlsxWriter) for writing Excel files. Note that, as time goes on, the package versions will be updated within the Google Colab environment.
